# Aneurism of the Right Subclavian Artery Treated by Direct Pressure on the Arteria Innominata

**Published:** 1867-12

**Authors:** 


### Aneurism of the right Subclavian Artery treated by direct Pressure on the Arteria Innominata.

In the *Medical Press and Circular* for July 3 and August 7, 1867, Mr. Porter, Surgeon to the Heath Hospital of Dublin, reports a case of aneurism involving the three portions of the subclavian artery. The patient had also another aneurism of the right femoral close to and passing above Poupart's ligament.

"As the subclavian aneurism was evidently thinning and threatening soon to become diffused or burst externally, Mr. Porter considered his a fair case to give him the chance (although unpromising) of a cure by occluding the artery on the distal side of the tumor. He therefore decided upon attempting to obliterate the aneurism by placing an acupressure needle under the axillary artery in its first stage for fifty hours. The general tendency to aneurism in his system induced Mr. Porter to prefer giving this mode of closing the vessel a trial, instead of throwing a ligature round it, which might, in the first instance, suddenly cut through the artery if diseased, or, when coming away, be followed by fatal hæmorrhage.

"June 26, 1867.—Mr. Porter laid bare the axillary artery in its first stage, after an external incision, four inches in length, extending in a curved direction inward, from the junction of the deltoid with the greater pectoral muscle, and at a level of half an inch below the clavicle. He then isolated the artery with an aneurism needle, and passed a silver probe slightly bent beneath it, and bridged over the vessel with a loop of wire, after the manner of Sir James Simpson's *third* mode of acupressure. The tumor immediately became reduced one-third in size, and all pulsation in his brachial and radial arteries ceased. The patient was then removed to bed, and a small bag of ice applied to the tumor."

On the 28th, the probe and wire were removed without hæmorrhage. The tumor was smaller and more firm, but the pulsations had not entirely ceased. The wound healed perfectly and the man was up and walking about in three weeks; but the pulsations gradually returned, and he became as strong as before the operation.

As the disease was increasing rapidly, Mr. Porter determined to attempt the cure by placing direct pressure on the innominate artery.

"July 31.—Mr. Porter laid bare the vessel after a tedious and careful dissection, which occupied nearly forty minutes. The operation he selected was almost similar to that performed by Mott. Instead of using the acupressure needle and wire, as on the former occasion, he employed a most ingenious instrument invented by Doctor L'Estrange, which somewhat resembles a double aneurism needle without eyes. It is furnished with a moveable handle. One blade is first carried under the vessel, and the second is then passed down on the artery, and is made to compress it like the manner in which the jaws of a lithotrite are closed, with the exception of the blades of this instrument being perfectly smooth and rounded. The blades are approximated by means of a screw, and when sufficiently brought together the handle is removed, leaving the needle in the wound. The patient bore the operation well, and all pulsation in the tumor was arrested by the instrument. He did not suffer the slightest inconvenience from shutting off the current of blood from that side of his head. A bag of ice was then applied to the tumor.

Aug. 2.—Mr. Porter removed the needle; no blood followed, but pulsation returned strongly in the aneurism.

The case is, we believe, the first on record in which occlusion of the innominata has been attempted by pressure without ligation.—*New York Medical Journal.*

---

Dr. J. J. Rooker, of Castleton, Marion Co., Ind., proposes to prepare some statistics, showing the effects of balls retained in the human body. Any one having cases of such injuries from fire-arms, and willing to report them, will be furnished with *blank forms* upon application to the doctor.

